# A Psychometric Study of the Student Evidence-Based Practice Scale S-EBPQ-Arabic Version for Use among Undergraduate Nursing Students

**DOI:** 10.1155/2024/6375596

**Published:** 2024-02-13

**Authors:** Basmah F. Alharbi

**Affiliations:** Department of Basic Health Science, College of Applied Medical Sciences, Qassim University, Buraydah 51452, Saudi Arabia

## Abstract

**Background:**

Previous studies have demonstrated the significance of evidence-based practice in improving patient care and outcomes. Therefore, integrating evidence-based practice into the health professions' education curriculum has become a pedagogical priority. However, there is a lack of reliable and valid scales to measure students' evidence-based practice usage, attitudes, knowledge, and skills in Arab countries.

**Aim:**

This study aims to examine the adapted Student Evidence-Based Practice Scale Questionnaire (S-EBPQ) validity at logical statistical level and reliability for use among students in Arabic context.

**Methods:**

This cross-sectional study included 233 undergraduate nursing students from a university in Saudi Arabia, who were recruited after translating and pilot testing the S-EBPQ. Three distinctive types of validity including conceptual, content, and face validity were assessed to determine the quality of the questionnaire items logically. Exploratory factor analyses were performed to examine the tool's structural validity. Additionally, internal consistency was assessed to evaluate reliability. *Findings*. All items were considered relevant to Arab culture, and no changes were made to any items. The content validity indices for all items were above 0.80 as this was considered an acceptable value. The exploratory factor analysis identified the same four factors (practice, attitude, retrieving and reviewing evidence, and sharing and applying evidence-based practice). All KMO values for the individual items ≥0.876 were also well above the acceptable 0.6 limit. The four-factor structure explained a total variance of 64%, with factor load score *λ* ≥ 0.455. The total and subscale S-EBPQ scores showed evidence of reliability, with Cronbach's alpha ≥0.8.

**Conclusions:**

This study demonstrated the reliability and validity of the Arabic S-EBPQ version. The study has the potential to advance Arab countries' understanding of evidence-based practice. S-EBPQ is a validated tool that can be used to assess nursing students' knowledge of EBP practices. Since educators need to continually evaluate instructional and curricular design in order to meet contemporary nursing needs, this scale can enhance the educational process and enhance students' competencies.

## 1. Introduction

Evidence-based practice (EBP) is defined as the thoughtful usage and integration of the best recent evidence, clinical expertise, and patient preferences to improve clinical care practice decisions [1, 2]. The EBP competencies in healthcare provide clarity and are a guide for clinicians, leaders, faculty, EBP mentors, and students as they strive to achieve EBP competencies. This is crucial for healthcare professionals to provide patients with the most efficient and effective care possible [3]. The use of EBP can enhance the quality, dependability, and outcomes of healthcare. In 2014, Melnyk et al. aimed to establish EBP competencies for nurses and advanced practice nurses in clinical settings. The competencies were initially formulated by experts in EBP and were subsequently refined through a Delphi survey, which engaged the input of 80 EBP mentors from various locations across the United States [4]. The survey identified 13 competencies for nurses and 11 for advanced practice nurses, providing consensus and clarity around the competencies [4]. The study recommended that to improve healthcare quality, reliability, and consistency while also reducing costs, healthcare system requirements, job descriptions, performance evaluations, orientation programs, and career advancement criteria should include these competencies [4]. Achieving evidence-based decision-making in daily practice and accelerating the translation of research knowledge into real-world settings are vital for improving health outcomes and lowering healthcare costs [5]. This can help reduce medical errors and improve patient outcomes. Furthermore, it encourages innovation in healthcare delivery, enabling healthcare providers to identify and implement new and better ways of providing healthcare [6].

Worldwide, nursing authorities emphasize the importance of aligning contemporary nursing practice with EBPs to enable the nursing discipline to address the gap between theory and practice [7–9]. EBP is built on four essential factors: first, the practice aspect focuses on the frequency of EBP use in clinical settings. Second, the attitude aspect focuses on personal judgments on EBP, which is crucial for the organizational culture to implement EBP [8, 9]. The third and fourth aspects focus on students' skills related to the identification and evaluation of evidence, application of evidence to specific cases, and dissemination of EBP knowledge. Globally, as recommended by the World Health Organization, healthcare delivery systems must maintain practice based on the best available EBP [10]. EBPs play a pivotal role in ensuring excellence in healthcare delivery. They contribute to the minimization of healthcare costs, help in the optimization of patient safety and health outcomes, provide a framework that aids clinical decision-making, and ultimately elevate the overall quality of care [8, 11]. Furthermore, EBPs help caregivers demonstrate professionalism, which is indispensable for promoting the growth of professional identity [11]. Approximately, 55% of all nursing practices are based on research findings [12].

The application of evidence in clinical practice is limited [13]. For example, the nursing literature has expanded its scope to examine EBP teaching strategies in nursing programs—particularly at the undergraduate level—to prepare undergraduate student nurses for professional practice and foster an appreciation of EBP [14, 15]. Emphasizing the competency and readiness of nursing students in implementing EBP is considered a priority because positive attitudes toward EBP are vital in the use of evidence in clinical practice [16, 17]. However, nursing students may be disinterested or harbor negative attitudes toward EBP [18, 19]. Incorporating EBP into the theoretical and practical components of nursing education can facilitate students' understanding and appreciation of EBP, which will ultimately assist them in successfully applying the evidence to clinical practice. Therefore, nursing educators require a valid measuring tool for assessing the EBP competencies of nursing students and understanding nurses' knowledge, attitudes, and skills in engaging with EBP. Furthermore, the tool is necessary for assessing the effectiveness of their teaching approaches for EBP [3]. However, in Arab countries, particularly Saudi Arabia, research instruments in the Arabic language for assessing student EBP competencies are limited. This gap could be attributed to the lack of psychometrically tested measures for use in Arab countries.

Florence Nightingale pioneered the use of scientific evidence to establish appropriate healthcare practices to provide safe and effective care [9]. In the last two decades, numerous researchers have delved into the exploration of EBP within the healthcare domain, leading to a substantial body of literature specifically dedicated to evidence-based nursing [20, 21]. However, despite academic interest and extensive literature on evidence-based nursing, the application of the best available scientific evidence in practice is lacking [13]. Various studies have highlighted the challenges and barriers faced by registered nurses when implementing EBP [22, 23]. Common difficulties include time and effort constraints and issues with critical appraisal, leading to biased use of evidence [24]. Nursing schools face the task of modifying their teaching and training methods to overcome the disparity between research and the actual application of nursing education in real-life settings.

Education is crucial for minimizing the gap between knowledge and practice; thus, concerns related to EBP must be addressed in healthcare education to develop student competencies in the early stages. Therefore, health science colleges should incorporate EBP into their theoretical and clinical curricula to ensure that frontline prospective caregivers are prepared to practice with appropriate knowledge and skills. To accomplish this, faculty should develop curricula that promote research and the application of scientific evidence because students will be able to acquire the necessary research competencies during their education, allowing them to develop scientific evidence for use in practice [25]. Thus, it becomes increasingly important for education programs to be continuously evaluated based on the use of a combination of teaching and learning methods and incorporate technology, while considering the learning styles of students and access to technology, which enhances both students' skills in solving questions using the current literature and databases and their attitudes toward EBP [13].

Recent trends in EBP have resulted in the development of several instruments that can be used to assess EBP implementation effectiveness. However, note that most instruments are irrelevant to students and were designed primarily for use by healthcare professionals, including registered nurses [26]. Because of the lack of instruments for assessing the acquisition of EBP competencies [27, 28], two instruments have been developed specifically for nursing students: the EBP Evaluation Competence Questionnaire (EBP-COQ) [25] and the Student Evidence-Based Practice Questionnaire (S-EBPQ) [15, 29]. The S-EBPQ was developed by adapting the EBPQ to measure nursing students' understanding and application of EBP at all levels of practice and was found to be valid and reliable [15, 16, 29]. The EBP-COQ is a reliable and valid 25-item instrument developed in Spain for undergraduates [25]. The two instruments are designed to assess nursing students' level of competency in EBP; however, they differ in the way EBP is conceptualized and operationalized. The S-EBPQ encompasses assessments of attitude, knowledge, skills, and frequency of use, whereas the EBP-COQ focuses solely on evaluating attitude, knowledge, and skills related to EBP. The S-EBPQ focuses on an individual's ability to apply evidence-based knowledge to clinical practice, whereas the EBP-COQ focuses on the individual's ability to assess and evaluate evidence [30]. Because EBP is about action [31], the frequency of use is crucial in assessing EBP. Therefore, the EBPQ has been adopted by various healthcare professionals, including dietitians, midwives, physiotherapists, speech therapists, and occupational therapists [32]. Among health professionals, this tool can be used to assess educational programs or policy developments [33]. This fact reinforces the universality and applicability of the EBPQ in health profession education. This is an invaluable tool for determining which students are competent in practicing EBP. As a result of the data collected from this tool, curriculum development can be influenced, and nursing students will be adequately prepared to serve as healthcare professionals. Furthermore, this instrument can be used to assess the effectiveness of current EBP teaching strategies.

In Saudi Arabia, the role of EBP in providing high-quality healthcare has recently been widely discussed [34]. In Saudi clinical settings, nurses have reported encountering various barriers to implementing EBP. These challenges include a lack of familiarity with the S-EBPQ and difficulties in comprehending the tool [34, 35]. The implementation of EBP by nursing students in the Saudi Arabian context is very low; therefore, training the next generation of nurses in EBP and focusing on their knowledge, attitudes, and skills are important [35]. This can be addressed by using robust measures for EBP. The existing measures have been tested on students globally and translated into different languages, such as Chinese and Korean [16, 36]. In particular, the EBPQ has been translated and validated in more than 25 languages. This makes the S-EBPQ a powerful tool for research and assessment across different cultures, countries, and regions. Unfortunately, the S-EBPQ is not available in Arabic. However, the S-EBPQ has only been statistically validated and is not conceptually validated, and it is in English, which is not the mother language of Saudi Arabia [37].

Conceptual content and face validity to evaluate the quality of each questionnaire item based on the item's contents rather than its response format must be tested [38]. Across cultures, differences in perceptions and interpretation of health-related concepts and indicators may exist [39–41]. To minimize the risk of adopting unfamiliar or unrelated concepts to the target culture, conceptual equivalence must be assessed [39]. Otherwise, the results may lead to inaccurate and misleading assumptions [42]. To ensure that the adopted S-EBPQ is clear, precise, understandable, culturally fit, and translated into the language of the target population so that they can participate as non-English speakers, assessing the validity of the S-EBPQ in Arab contexts is necessary. The translation of the S-EBPQ into multiple languages has been useful, regardless of students' English proficiency. Furthermore, translating the S-EBPQ into the mother tongues of the participants serves many purposes. The first benefit of answering questions in one's native language is that one has less cognitive load when understanding and interpreting foreign language questions. Therefore, participants can answer questions more accurately by understanding their meanings and nuances. Furthermore, language proficiency varies from individual to individual, regardless of whether the individual has been educated in English. The Arabic translation of the S-EBPQ ensures that participants understand the questions and provide accurate and meaningful responses. Moreover, language is closely related to culture; therefore, using the participant's mother tongue is important to ensure cultural relevance.

This study addresses the lack of data on the psychometric properties of the Arabic version of the S-EBPQ in a cross-cultural setting, particularly in Saudi Arabia, for use among undergraduate students at health science colleges. Healthcare educators and professionals may benefit from this study's guidance on how to design and validate the S-EBPQ. This study aims to examine the validity and reliability of the adapted S-EBPQ at the logical statistical level and its suitability for use among students in an Arab context.

## 2. Methods

Strengthening the Reporting of Observational Studies in Epidemiology (STROBE) checklist [42] was followed reporting this research. The purpose of this reporting tool is to systematically check and evaluate the reliability of the research.

### 2.1. Design

A cross-sectional study was conducted in two phases. In phase one, the scale was conceptually validated and linguistically translated. In phase two, the scale was distributed to undergraduate students to evaluate the psychometric properties of the Arabic version of S-EBPQ.

### 2.2. Procedures and Samples

#### 2.2.1. Conceptual Validation and Translation Phase

Conceptual validation and linguistic translation were performed based on a standardized method [39]. This phase assessed the accuracy of the items in relation to the study culture before making a decision regarding translation. This assessment was made to avoid spending time on several revisions of the measure after translation and thus wasting resources. Conceptual validity was assessed based on comprehension and cultural relevance to participants' understanding. First, an expert panel of six panellists assessed item comprehension and cultural validity of S-EBPQ (English version) using Sidani et al. criteria [39]. As part of the selection process, two experts from each of the following groups were selected: (1) Health professionals' group, nurses, who are familiar with the challenges or issue of understating and implementing EBPQ and population culture; (2) nursing educators who are familiar with challenges that face students in placement settings; (3) laypersons' group, Saudi nursing graduated students. The members of the panel were Saudis, bilingual, and aware of the challenges and values of the studied group. Additionally, two recently graduated nursing students had experience in the challenges and the barriers that could face students in understating and evaluating the questionnaire items. The panel was thus fit for the study's purpose. Each panel member was requested to individually rate each item on a 10-point scale in terms of its conceptual validity. First, the comprehension domain was examined to assess whether the concepts of the items would be easily understandable in the Saudi nursing context. Second, the cultural relevance domain was examined to assess the relevance of the items' content to the Saudi nursing culture. The comprehension and cultural relevance indexes for items were calculated as the number of experts giving a score >5 for each item, divided by the total number of experts. As all items were considered relevant (the comprehension and cultural relevance indexes for all the items were 1.00), no changes were made to any item.

Following assessing conceptual validity, a forward translation of 21 items was performed by two bilingual translators. A backward translation was not conducted because there was a high level of agreement among the translators and considered an optional phase in the conceptual validation and translation of measures process [39]. The focus should be on conceptual and cultural equivalence rather than linguistic equivalence in translation [39].

#### 2.2.2. Content Validation Phase

After conceptual validation and translation, face and content validation were conducted in the following phases. Clearness and relevance of the translated items were assessed by experts, laypeople, and samples from the target population. In order to determine the level of quality of translated measures, especially in cross-cultural studies, it was necessary to assess the content validity (CV) of the S-EBPQ-Arabic version. The Arabic version of CV was assessed based on a 4-point Likert scale by eight experts who were not part of the conceptual validation process to determine the item-level content validity index (I-CVI) as follows: 1 = (not relevant), 2 = (somewhat relevant), 3 = (quite relevant), and 4 = (highly relevant). A relevance assessment would ensure that the translated items focus on the constructs within the S-EBPQ rather than on any broader concepts, ensuring a close fit between the translated and included items. Based on the recommendations of Rubio et al. [43], a panel of eight experts was established that included both content and lay experts, as well as methodologists and linguists. As part of the selection process, the following criteria were used: (1) topic experts, three bilingual nurses who have experienced clinical practice challenges in nursing; (2) methodologists, three bilingual nursing educators with experience designing questionnaires; and (3) two bilingual laypersons, nursing graduates, who are members of the target group. The I-CVIs for all items were above 0.80 as this was considered an acceptable value [43].

#### 2.2.3. Face Validation Phase

Face validation was conducted with a sample of 20 undergraduate nursing students who had completed the research course and had a clinical placement. Participants who did not meet these criteria were excluded from the study, such as those who did not complete a research course or a clinical placement. A sample size was determined based on the face validity evaluation phase as a pilot test. Pilot studies usually include 10–30 participants, depending on their particular purpose. In this phase, the translation tool was evaluated for its face validity. Although the existing instrument is approved and validated, a pretest is necessary to determine its length, clarity, and overall suitability [44]. Several aspects of the study were assessed, including the feasibility of the study, the clarity of the prefinal translation, and language appropriateness. The pilot test demonstrated that the items were clear and easily understandable.

#### 2.2.4. Psychometric Phase

A convenience sample of undergraduate nursing students was recruited in January 2019, from the King Saud University (KSU), which is a public university in Riyadh. The study included nursing students who met two criteria: completion of the research course and exposure to clinical placement during clinical rotations. Nursing students who did not meet these criteria, such as those who did not complete the research course or participate in a clinical placement or internship year, were excluded from the study.

Consequently, all participants possess the minimum skills required to develop PICO questions, search for relevant literature, and evaluate the results obtained. All potential participants were provided with a participant information sheet containing information about the study. Following an explanation of the study's requirements and purposes, students who agreed to participate signed an informed consent form and completed the questionnaire. The data collection was carried out using the paper-and-pencil format.

The sample size was calculated based on the size recommended by Nunnally, who suggested a ratio of ten cases per item [45]. Given that the scale has 21 items, a sample of 210 participants was considered sufficient for conducting the analysis. The response rate of the current study was 93% as 250 questionnaires were distributed, as this size would allow for missing data, 233 valid questionnaires were returned.

### 2.3. Instrument

The students answered questions related to their demographic characteristics (i.e., age, gender, nationality, and year of study) and questions relating to the acceptance and implementation of EBP by using the S-EBPQ. The S-EBPQ is a 21-item self-report questionnaire measured with a 7-point Likert scale with four subscales, practice, attitude, retrieving and reviewing evidence, and sharing and applying EBP. Six items for practice subscales from “never” to “frequently,” three items for the attitude subscale measured on a 7-point semantic differential scale from negative to positive, seven items for retrieving and reviewing evidence subscale measured from “poor” to “best” lastly five items for sharing and applying EBP subscale measured from “poor” to “best” [14]. The S‐EBPQ reliability and validity is valid and reliable tool [14, 15]. The S-EBPQ has reported good reliability, and the Cronbach's alpha scores for practice, attitude, retrieving and reviewing evidence, and sharing and applying EBP were 0.847, 0.765, 0.912, and 0.852, respectively [14].

### 2.4. Ethical Consideration

The required permissions were obtained by the authors prior to adapting and translating the scale. A registration is required to access the tool on the EBPQ website, as granted by the authors. The study was approved by the Institutional Review Board (IRB) (Approval No: KSU-E-19-2612) in 2019 before data collection. Informed consent was obtained from the participants after explaining that the study was voluntary, the data would remain confidential, they could withdraw from the study at any time, and that nonparticipation would not affect their grades. The questionnaires were returned without students' identification information, and each participant received an anonymized ID. Anonymity was ensured to minimize the bias related to social desirability.

### 2.5. Statistical Analysis

The analyses were carried out in steps using SPSS version 21 and Mplus 7 [46]. Inspection of the data showed that there were no missing data. Descriptive statistics (frequencies and percentages) were analysed to describe the sample demographic characteristics. The factorial analysis, Cronbach's alpha, and bivariate correlations were conducted to assess the S-EBPQ psychometric properties.

Exploratory factor analysis (EFA) was conducted to ascertain the construct validity of the underlying constructs of the Arabic version of the S-EBPQ. First, an exploratory factor analysis (EFA) with principal axis factoring and oblique rotation was conducted to test the factor structure. Eigenvalues, the percent of variance accounted for by each extracted factor, and the theoretical salience of the rotated factors were inspected to select the best model (number of factors). A threshold of 0.4 was used to differentiate the factor loading.

Second, the internal consistency reliability was assessed for the four subscales (practice, attitude, retrieving and reviewing evidence, and sharing and applying EBP). Both the interitem correlation and correlations of items with their factors were assessed to support the reliability findings [47] using the suggested thresholds ranging from 0.30 to 0.70 for interitem correlations, and above 0.30 for corrected item total correlations [44, 47].

## 3. Results

### 3.1. Participant Demographics

As presented in Table 1, around 49.3% of the students in the program were 22 years old, while 50.6% were 23 years old. The majority of the students, about 97.9%, were Saudi nationals, and Arabic was the language spoken by both Saudis and non-Saudis. Out of the total number of students, approximately 54.5% were male. Among all the students, 61% were in their fourth year of the program, while 39.1% were in the internship year of the program.

### 3.2. Factor Analysis

EFA using a principal axis factoring (PAF) estimation and oblique rotation was conducted to identify the factor structure for the S-EBPQ. The PAF method of factor extraction was used as it analyses common variance, and oblique rotation was chosen as it allows factors to covary [48]. A four-factor solution was identified as the best solution (first five eigenvalues: 7.32, 3.16, 1.97, 1.28, and 0.92).

The KMO measure confirmed the appropriateness for analysis at KMO = 0.876, exceeding the recommended value of 0.6. Additionally, Bartlett's sphericity reached a statistical significance of *p* < 0.001, supporting the factor ability of the correlation matrix [47, 49]. Based on Kaiser's criterion, a 4-factor structure was extracted, which explained 64% of the variance. Each factor contributed a percentage of the explained variance as follows: factor 1 (practice explained variance of 34%), factor 2 (attitude explained variance of 15%), factor 3 (retrieving and reviewing evidence explained variance of 9%), and factor 4 (sharing and applying EBP explained variance of 6%). Nineteen of the twenty-one items were loaded cleanly onto one of the four factors. Two items (17 and 18) loaded significantly on two factors. However, both items were retained under factor 4, similar to Upton et al.'s original structure, based on the highest loading [14]. The labels of the four-factor structure suggested by Upton et al. were maintained [14]. The final rotating factor loadings on labelled factors are shown in Table 2. The four factors reflect unique constructs as they were moderately correlated, ranging from 0.21 to 0.67 (Table 3). These moderate correlations (<0.85) support the scale's construct validity [47–49].

### 3.3. Item Analysis and Reliability

The descriptive statistics for all items were assessed, and they were normally distributed (Table 4). The item total correlations ranged from 0.380 to 0.679. The mean interitem correlation coefficient for the entire S-EBPQ was 0.321. For the practice subscale, the item total correlations ranged from 0.603 to 0.709, with the interitem correlations ranging from 0.383 to 0.596. For the attitude subscale, the item total correlations ranged from 0.604 to 0.645, with the interitem correlations ranging from 0.680 to 0.712. For retrieving and reviewing evidence subscale, the item total correlations ranged from 0.610 to 0.721, with the interitem correlations ranging from 0.367 to 0.672. For sharing and applying EBP subscale, the item total correlations ranged from 0.681 to 0.780, with the interitem correlations ranging from 0.401 to 0.724. The internal consistency of the S-EBPQ was 0.897 for the full scale, 0.859 for the practice subscale, 0.833 for the attitude subscale, 0.878 for retrieving and reviewing the evidence subscale, and 0.891 for the sharing and applying EBP subscale.

### 3.4. Students' Practice, Attitudes, and Knowledge Related to EBP

On a Likert scale from 1 to 7, the results demonstrate that 79.9% of the participants responded that they “occasionally to always” appraised literature using the set of criteria, 82.5% “occasionally to always” evaluated the outcome of their practice, 84.1% “occasionally to always” shared research information with colleagues, 83.2% welcomed questioning of their clinical practices, 78.5% reported that EBP is fundamental to professional practice, 69.4% rated their research skills as “good to excellent,” 61.9% rated their ability to convert needed information into research questions as “good to excellent.” Overall, 67.4% of the participants reported that they frequently practiced EBP, 82.4% reported having a positive attitude toward EBP, 46.8% had excellent levels of retrieving and reviewing evidence, and 70% had exceptional levels of sharing and applying EBP. The students' scores on each subscale and the total are presented in Table 5.

## 4. Discussion

The purpose of this study was to translate and test the reliability and validity of the S-EBPQ in a sample of undergraduate nursing students in Saudi Arabia. The content validity was evaluated by experts, and the construct validity was assessed by exploratory factor analysis; both were confirmed by the results. Thereafter, the reliability was tested based on internal consistency. This study contributes to existing knowledge by replicating and testing the S-EBPQ instrument in an Arab context. This is also the first conceptual and construct validation of an Arabic EBP scale. As a result of the study, it was found that the scale is a reliable and valid tool for assessing four distinct dimensions of EBP (practice, attitudes, retrieving and reviewing evidence, and sharing and applying evidence). Students' responses in validation phase indicated that the S-EBPQ was easy to use and understand, and that it was effective for implementing EBP.

Saudi Arabia's nursing research curriculum and EBPs might differ from Western countries [50]. However, the study found that the S-EBPQ was a perfect measure of EBP competency in Saudi Arabia and there were no cultural differences to be noted. This suggests that, despite cultural differences, EBP principles are still relevant and applicable to Saudi Arabia. This is supported by the fact that EBPQ is a universal tool that has been translated and validated into over 25 different languages. This makes the S-EBPQ a powerful tool for research and assessment across a number of cultures, countries, and geographical areas. Having conducted conceptual, content, and face validations as well as EFA and reliability, this study has confirmed that the tool is culturally valid in the Saudi context. Consequently, the Arabic scale may contribute to the expansion of research on assessing EBP among nursing students in Arab countries as a result of its availability. Students' EBP competency can be assessed with the S-EBPQ, and it can be used to compare nursing education programs. Furthermore, it can be used to identify areas for improvement and to guide student learning.

The study's findings are consistent with those of previous studies as it demonstrated the reliability and validity of the translated S-EBPQ-Chinese version [16], S-EBPQ-Korean version [36], and untranslated S-EBPQ-English version [14, 35, 37]. The internal consistency of the Arabic version of the S-EBPQ was 0.897 across the entire scale. The coefficient alphas of the four subscales were above 0.80; thus, the internal consistency reliability was good [44]. The EFA suggested that the translated scale is similar to the original S-EBPQ with the majority of the items loaded on the intended subscales. Only items 17 (ability to identify gaps in your professional practice) and 18 (ability to apply information to individual cases) cross loaded on two subscales. However, both of these items loaded more strongly on the sharing and applying EBP subscale. Thus, it is similar to the original structure of Upton et al. [14]. In addition, the four-factor structure explained 64% of the variance, which is similar to the original scale that explained 65% of the variance [14], while the S-EBPQ-English version in Saudi context explained 62% of the total variance [37].

The estimated correlations among the factors were not extremely high, which supports the construct validity [46–48]. The following four factors are identified as important aspects to assess among students: practice, attitudes, retrieving and reviewing evidence, and sharing and applying evidence. Education utilizing this tool could ensure that future generations of nurses have the required skills to implement EBP. It is recommended that the S-EBPQ scale be utilized to assess undergraduate students' EBP to gain a better understanding of current education programs.

The students in the current study have clinical placements as part of their program; therefore, it is essential for nursing educators to continually assess and invest in preparing nursing students to implement EBP. EBP must be well integrated into the curriculum to ensure that future nurses are adequately prepared for contemporary practice and increase the quality of healthcare. The current sample of nursing students showed the required level of EBP implementation in their clinical practice. They are at the point of identifying the existing gaps in practice, as well as applying particular information.

### 4.1. Limitations

While the study provides valuable insights into the psychometric properties of the revised cross-cultural S-EBPQ tool, several limitations should be acknowledged. Firstly, construct validation was limited to exploratory factor analysis (EFA) and confirmatory factor analysis (CFA) was not conducted. Future studies should incorporate CFA to further enhance the validity of the scale across diverse cultural contexts. Additionally, reliability assessment was confined to internal consistency, overlooking test-retest reliability and interrater agreement. Furthermore, the study did not explore criterion validity due to constraints in time, financial resources, and logistical challenges. The sequential completion of multiple validation steps and the involvement of various stakeholders with different expertise levels posed challenges.

The generalization and applicability of the Arabic S-EBPQ may be constrained as the study focused on a nonrepresentative sample from a single public university. While efforts were made to include students from different sociocultural backgrounds within the university, future research should consider diverse samples from multiple institutions to enhance generalizability. The use of convenience sampling, though practical, may limit generalizability; thus, future studies could explore alternative sampling methods. Moreover, despite a high response rate (93%), potential sampling bias cannot be entirely ruled out.

### 4.2. Future Research Directions

In light of the identified limitations, future research endeavours can enhance the current study's findings and contribute to the broader field of evidence-based practice in nursing education. Subsequent investigations should prioritize the inclusion of confirmatory factor analysis to confirm the scale's validity across cultures rigorously. Exploring test-retest reliability and interrater agreement will provide a more comprehensive understanding of the scale's stability over time and consistency across different raters.

Additionally, future studies could delve into criterion validity by examining the relationship between the S-EBPQ scores and external criteria relevant to evidence-based practice. This would further establish the scale's effectiveness in measuring what it intends to measure. To overcome logistical challenges, researchers might consider collaborative efforts with multiple institutions and leverage technological advancements for data collection.

Furthermore, investigating the applicability of the Arabic S-EBPQ among diverse health sciences student populations can enrich our understanding of evidence-based practice education across different disciplines. Comparative studies across institutions and regions can elucidate variations in students' perceptions and engagement with evidence-based practice. Finally, longitudinal studies can provide insights into the long-term stability and effectiveness of the S-EBPQ in tracking students' evolving attitudes and skills throughout their education and into their professional practice.

### 4.3. Impact Statement

The Arabic version of the S-EBPQ has been culturally adapted and linguistically translated, and its psychometric properties were tested. The use of this scale can enhance EBP education and competencies. Nursing educators are required to continually evaluate instructional and curricular design to better meet the needs of contemporary nursing. This evaluation requires assessing nursing students' EBP competencies using a validated tool, such as S-EBPQ.

## 5. Conclusion

The importance of EBP has been emphasized worldwide; however, there is a lack of reliable and valid measures that assess the acceptance and implementation of EBP among students in the Arab world. This study revealed that the Arabic version of the S-EBPQ is a reliable and valid measure. It assesses EBP practices, attitudes, knowledge, and skills in nursing students. Therefore, this version has the potential to evaluate undergraduate nursing students' engagement with EBP in the Arab population and could be used by institutions to understand students' learning needs. However, further testing of the Arabic S-EBPQ with other samples and other health sciences students are needed.

## Figures and Tables

**Table 1:** Participants' demographic characteristics.

	Frequency	Percent (%)
*Age*		
22	115	49.3
23	52	50.6

*Gender*		
Male	127	54.5
Female	106	45.5

*Nationality*		
Saudi	228	97.9
Other	5	2.1

*Study level*		
Fourth year	142	61
Internship year	91	39.1

**Table 2:** Standardized factor loadings for the Arabic S‐EBPQ based on EFA using principle axis factoring.

Items	Factor 1 (practice)	Factor 2 (attitude)	Factor 3 (retrieving and reviewing evidence)	Factor 4 (sharing and applying EBP)
Q1. Formulated a clearly answerable question	**0.686** ^*∗*^	0.075	0.041	0.015
Q2. Tracked down the relevant evidence	**0.745** ^*∗*^	0.002	0.019	0.104
Q3. Critically appraised, against set criteria	**0.751** ^*∗*^	0.070	0.119	0.075
Q4. Integrated the evidence	**0.722** ^*∗*^	0.012	0.120	0.047
Q5. Evaluated the outcomes of your practice	**0.670** ^*∗*^	0.077	0.136	0.199
Q6. Shared this information with colleagues	**0.577** ^*∗*^	0.109	0.176	0.298
Q7. I resent having my clinical practice questioned	0.070	**0.776** ^*∗*^	0.096	0.084
Q8. Evidence-based practice is a waste of time	0.038	**0.822** ^*∗*^	0.068	0.097
Q9. I stick to tried and trusted methods	0.021	**0.781** ^*∗*^	0.010	0.030
Q10. Research skills	0.010	0.024	**0.701** ^*∗*^	0.081
Q11. Converting your information needs	0.036	0.021	**0.675** ^*∗*^	0.010
Q12. Awareness of major information types	0.021	0.077	**0.629** ^*∗*^	0.157
Q13. Knowledge of how to retrieve evidence	0.103	0.100	**0.740** ^*∗*^	0.039
Q14. Ability to analyse critically	0.153	0.055	**0.711** ^*∗*^	0.115
Q15. Ability to determine how valid (close to the truth) the material is	0.006	0.025	**0.680** ^*∗*^	0.160
Q16. Ability to determine how useful (clinically applicable) the material is	0.010	0.031	**0.632** ^*∗*^	0.222
Q17. Ability to identify gaps	0.061	0.037	0.310^*∗*^	**0.470** ^*∗*^
Q18. Ability to apply information	0.041	0.162	0.361^*∗*^	**0.455** ^*∗*^
Q19. Sharing of ideas and information with colleagues	0.039	0.020	0.088	**0.838** ^*∗*^
Q20. Dissemination of new ideas	0.056	0.032	0.160	**0.703** ^*∗*^
Q21. Ability to review your own practice	0.012	0.162	0.237	**0.626** ^*∗*^

Items with the factor load score *λ* ≥ 0.4.

**Table 3:** Correlation matrix for the four factors.

Factors	1	2	3	4
(1) Practice subscale	1			
(2) Attitude subscale	0.39	1		
(3) Retrieving and reviewing evidence subscale	0.26	0.21	1	
(4) Sharing and applying EBP subscale	0.37	0.40	0.67	1

**Table 4:** Item-level descriptive statistics of the S‐EBPQ (*N* = 233).

Items	Mean	SD	Skewness	Kurtosis	Corrected item total correction
(1) Formulated a clearly answerable question	4.64	1.77	−0.56	−0.34	0.401
(2) Tracked down the relevant evidence once you have formulated the question	4.58	1.78	−0.58	−0.40	0.399
(3) Critically appraised, against set criteria any literature you have discovered	4.78	1.73	−0.53	−0.37	0.536
(4) Integrated the evidence you have found with your expertise	4.87	1.84	−0.69	−0.45	0.503
(5) Evaluated the outcomes of your practice	5.09	1.86	−0.89	−0.18	0.488
(6) Shared this information with colleagues	5.27	1.77	−0.87	−0.14	0.492
(7) I resent having my clinical practice questioned	5.94	1.57	−1.54	2.34	0.436
(8) Evidence-based practice is a waste of time	5.84	1.66	−1.21	1.62	0.380
(9) I stick to tried and trusted methods rather than changing to anything new	5.56	1.72	−0.14	0.74	0.402
(10) Research skills	4.33	1.40	−0.14	−0.17	0.427
(11) Converting your information needs into a research question	4.21	1.40	0.20	−0.37	0.504
(12) Awareness of major information types and sources	4.62	1.50	−0.11	−0.60	0.579
(13) Knowledge of how to retrieve evidence	4.42	1.53	−0.06	−0.70	0.528
(14) Ability to analyse critically evidence against set standards	3.94	1.23	−0.09	0.78	0.487
(15) Ability to determine how valid (close to the truth) the material is	4.60	1.39	−0.16	−0.45	0.563
(16) Ability to determine how useful (clinically applicable) the material is	4.79	1.54	−0.19	−0.67	0.560
(17) Ability to identify gaps in your professional practice	4.74	1.45	−0.35	−0.32	0.651
(18) Ability to apply information to individual cases	4.87	1.42	−0.47	−0.23	0.679
(19) Sharing of ideas and information with colleagues	5.29	1.51	−0.72	−0.01	0.639
(20) Dissemination of new ideas about care to colleagues	5.05	1.47	−0.47	−0.44	0.594
(21) Ability to review your own practice	5.22	1.55	−0.69	−0.15	0.658

**Table 5:** Students' scores related to EBNP.

S-EBPQ subscale	Median	Mean	SD	Range	Min–max
Practice	30	29.23	8.21	36	6–42
Attitude	19	17.33	4.29	18	3–21
Retrieving and reviewing evidence	31	30.91	7.59	42	7–49
Sharing and applying EBP	26	25.18	6.17	30	5–35
EBPQ total scale score	105	102.65	19.00	124	21–145

## Data Availability

The data that support the findings of this study are available from the corresponding author, Basmah F. Alharbi, upon reasonable request.
